# Targeted Depletion of Primary Cilia in Dopaminoceptive Neurons in a Preclinical Mouse Model of Huntington’s Disease

**DOI:** 10.3389/fncel.2019.00565

**Published:** 2019-12-20

**Authors:** Rasem Mustafa, Grzegorz Kreiner, Katarzyna Kamińska, Amelia-Elise J. Wood, Joachim Kirsch, Kerry L. Tucker, Rosanna Parlato

**Affiliations:** ^1^Institute of Applied Physiology, University of Ulm, Ulm, Germany; ^2^Institute of Anatomy and Cell Biology, Medical Cell Biology, University of Heidelberg, Heidelberg, Germany; ^3^Department of Brain Biochemistry, Maj Institute of Pharmacology, Polish Academy of Sciences, Kraków, Poland; ^4^Department of Pharmacology, Maj Institute of Pharmacology, Polish Academy of Sciences, Kraków, Poland; ^5^Jagiellonian Center for Experimental Therapeutics, Jagiellonian University, Kraków, Poland; ^6^Department of Biomedical Sciences, Center for Excellence in the Neurosciences, College of Osteopathic Medicine, University of New England, Biddeford, ME, United States

**Keywords:** primary cilium, dopamine system, Huntington’s disease, mTOR, p62

## Abstract

Multiple pathomechanisms triggered by mutant Huntingtin (mHTT) underlie progressive degeneration of dopaminoceptive striatal neurons in Huntington’s disease (HD). The primary cilium is a membrane compartment that functions as a hub for various pathways that are dysregulated in HD, for example, dopamine (DA) receptor transmission and the mechanistic target of rapamycin (mTOR) pathway. The roles of primary cilia (PC) for the maintenance of striatal neurons and in HD progression remain unknown. Here, we investigated PC defects in vulnerable striatal neurons in a progressive model of HD, the mHTT-expressing knock-in zQ175 mice. We found that PC length is affected in striatal but not in cortical neurons, in association with the accumulation of mHTT. To explore the role of PC, we generated conditional mutant mice lacking IFT88, a component of the anterograde intraflagellar transport-B complex lacking PC in dopaminoceptive neurons. This mutation preserved the expression of the dopamine 1 receptor (D1R), and the survival of striatal neurons, but resulted in a mild increase of DA metabolites in the striatum, suggesting an imbalance of ciliary DA receptor transmission. Conditional loss of PC in zQ175 mice did not trigger astrogliosis, however, mTOR signaling was more active and resulted in a more pronounced accumulation of nuclear inclusions containing mHTT. Further studies will be required of aged mice to determine the role of aberrant ciliary function in more advanced stages of HD.

## Introduction

Huntington’s disease (HD) is an autosomal dominant progressive neurodegenerative disorder caused by the toxic expansion of CAG trinucleotide repeats at the N-terminus of the Huntingtin gene. The mechanisms underlying selective vulnerability of dopaminoceptive medium spiny neurons (MSNs), resulting in impaired control of voluntary movement in HD, remain elusive (Ghosh and Tabrizi, 2018). The variability of disease onset and progression depends on the CAG number, and on genetic modifiers interacting with the Huntingtin mutation [Genetic Modifiers of Huntington’s Disease (GeMHD) Consortium (2015)]. Multiple signaling pathways and cellular functions are affected by mutant Huntingtin (mHTT), including protein aggregate degradation, which results in the accumulation of toxic proteins (Saudou and Humbert, 2016).

Primary cilia (PC) are single, non-motile microtubule-based organelles resembling a cellular antenna that represents a hub for receptors and components of numerous signaling pathways (Malicki and Johnson, 2017). Lack of HTT results in reduced and aberrant PC growth, and increased mHTT results in increased ciliogenesis (Keryer et al., 2011). Notably, longer PC have been observed in immortalized cellular models of HD in culture, and ependymal cilia in the lateral ventricles are disorganized in a mouse model of HD and in HD human post-mortem brains (Keryer et al., 2011). Another study showed that photoreceptor cilia pathology accounts for their degeneration in the retina of R6/2 transgenic mice overexpressing exon 1 of the human mHTT (Karam et al., 2015). It has been previously proposed that PC altered structure might affect the function of signaling pathways whose components are localized in the PC (Maiuri et al., 2013; Kaliszewski et al., 2015).

Interestingly, increased PC length results in the induction of autophagy by inhibition of the mechanistic target of rapamycin (mTOR) kinase activity (Kaliszewski et al., 2015), and components essential for ciliogenesis are degraded by autophagy (Pampliega et al., 2013; Tang et al., 2013). Because autophagy is altered in HD (Ravikumar et al., 2004), as well as dopamine (DA)-mediated signaling (Chen et al., 2013), it is possible that HD pathophysiology depends, at least in part, on defective cilia. These previous studies investigated neither PC dysfunction in the most vulnerable striatal neurons, nor the impact of defective PC on HD pathogenesis in the striatum. A deeper understanding of the role of PC in mHTT-dependent neurotoxicity might help to identify new determinants modifying HD progression.

To this end, we monitored neuron- and stage-specific changes of PC structure in a full-length progressive mouse model of HD, called zQ175 (Menalled et al., 2012; Carty et al., 2015). This knock-in model carries a chimeric human/mouse *HTT* exon 1 containing expanded CAG repeats within the murine *htt* gene and recapitulates several hallmarks of HD pathology (Menalled et al., 2012; Farrar et al., 2014; Carty et al., 2015; Ma et al., 2015). Moreover, we generated a new genetic mouse model of defective ciliary function in striatal neurons, as a tool to investigate the specific impact of PC loss on striatal neuron maintenance and on HD neuropathological hallmarks.

## Materials and Methods

### Mice

To generate a mutant mouse in which the *Ift88* gene is conditionally ablated by the Cre-LoxP system in MSNs, we employed the B6.FVB/N-Tg(D1RCre)Gsc (D1R:Cre) transgene, which expresses the Cre recombinase under the control of the dopamine 1 receptor (D1R) promoter (Lemberger et al., 2007). The D1R:Cre mice were crossed to *Ift88*^tm1.1Bky^ mice carrying the *Ift88* floxed allele (Ift88^*flox/flox*^; Haycraft et al., 2007) to generate *Ift88*^flox/flox^; D1R:Cre mice (Ift88^*D1RCre*^; here abbreviated *Ift88* cKO mice) that lack PC in MSNs. Htt^tmtm1Mfc^/190tChdi (zQ175 knock-in) mice were received courtesy of the CHDI Foundation from the Jackson Laboratory. The analysis of the genotype was performed by PCR of tail snips as previously described (Levine et al., 1999). For the experiments reported here, male and female mice were used and wild-type and mutant littermates were analyzed. The zQ175 knock-in mice carry ca. 190 CAG repeats in a chimeric human/mouse exon 1 of the murine huntingtin gene (Menalled et al., 2012). The zQ175 mutation was kept in heterozygosity, to limit toxicity and mimic a genetic situation more relevant for the disease, as it is autosomal dominant (Menalled et al., 2012). These mutant mice were born at the expected Mendelian ratio; they showed normal lifespan and no gross abnormalities (monitored until 1-year-old).

For genotyping of D1R:Cre and floxed *Ift88* alleles by PCR the following primer pairs was used: forward-primer/Cre (5′-GGA AAT GGT TTC CCG CAG AAC-3′) and reverse-primer/Cre (5′-ACG GAA ATC CAT CGC TCG ACC-3′), BY919 (5′-GGTCCTAACAAGTAAGCCCAGTGTT-3′) and BY598 (5′-GCCTCCTGTTTCTTGACAACAGTG-3′), respectively. For the zQ175 we used forward-primer/Neo: 5′-GAT CGG CCA TTG AAC AAG ATG– 3′ and reverse-primer/Neo: 5′-AGA GCA GCC GAT TGT CTG TTG– 3′.

### Brain Dissection and Tissue Preparation

For histological analysis, brains were either transcardially perfused or post-fixed in 4% paraformaldehyde (PFA; pH 7.2) overnight at 4°C. After washing in PBS (pH 7.2) the brains were cryoprotected by incubating them in 10%, 20%, and 30% sucrose for 3 days at 4°C. The brains were embedded in a coronal orientation (Tissue freezing medium, Leica), frozen by a mixture of liquid nitrogen and dry ice, and stored at −80°C until sectioning (Leica CM3050S cryostat). For the analysis of adult brains, we have used coronal sections from striatum collected serially on Superfrost Ultraplus glass slides (12 μm) and free-floating striatum (30 μm). We analyzed the striatum in the region comprised between Bregma +1.18 mm and −0.34 mm based on the adult mouse brain Atlas (Franklin, 2008). For immunofluorescence on paraffin sections, one brain hemisphere was fixed in 4% PFA in PBS, pH 7.2 overnight at 4°C and paraffin-embedded.

### Immunofluorescence

Immunofluorescence (IF) on cryosections was performed according to established protocols (Gazea et al., 2016). Cryosections (12 μm) were pre-incubated with 5% normal pig serum in PBS for 30 min at room temperature before overnight incubation at 4°C with the primary antibodies appropriately diluted in the blocking solution. After washing in PBS the sections were incubated with the secondary antibodies (diluted in 5% pig serum in PBS) for 30 min at room temperature. After further washes in PBS the sections were stained for 10 min with 4′,6′-diamidino-2-pheylindol (DAPI, Thermoscientific) diluted in PBS.

Free-floating cryosections (30 μm) were treated in a similar way; however, PBST (0.2% Triton X-100 in PBS) was used for washing steps and blocking solution. After staining the sections were placed on glass slides. Paraffin sections (7 μm) containing the striatum comprised between Bregma +0.14 mm and −0.98 mm were incubated with primary antibodies overnight at 4°C. Visualization of antigen-bound primary antibodies was carried out following antigen retrieval (HK086-9K, Biogenex). The following primary antibodies were used: EM48 (1:100, MAB5374, Millipore), NeuN (1:100, MAB377, Millipore), adenylate cyclase III (ACIII; 1:500, SC588, Santa Cruz Biotechnology, Dallas, TX, USA), TH (1:500, AB1542, Millipore), D1R (1:500, D2944, Sigma), glial fibrillary acidic protein (GFAP; 1:300, G-3893, Sigma), phospho-S6 (S235/236; 91B2; 1:100, 4857S, Cell Signaling), p62/SQSTM1 (1:100, P0067, Sigma). Incubation with the appropriate secondary antibodies marked with the fluorophores Alexa 594 or Alexa 488 (1:100, Thermoscientific) was followed by DAPI staining.

### Confocal Microscopy Imaging and Image Analysis

Images were acquired by a Leica SP8 confocal and a Leica LASX software. For PC number and length quantification, we used a 63× oil-immersion objective maximal intensity projection z-stacked images (1 μm interval). To determine the number of striatal NeuN positive neurons showing PC stained by ACIII antibody eight non-consecutive free-floating cryosections per mouse were analyzed (one every fourth section, each 30 μm thick) in control and mutant mice. Either the number or the length of PC per microscopic field at 63× magnification was counted. In general, about 80 cells per mouse were measured by the ImageJ software after tracing the ACIII signal in line with established protocols (Miyoshi et al., 2014; Parker et al., 2016). For semi-quantitative analysis of D1R and TH immunoreactivity, and of GFAP and phosphoS6 positive cells, we used a 20× oil-immersion objective for single planes. For p62 positive cells, we used a 63× and for the EM48 a 100× oil-immersion objective. To determine D1R and TH immunoreactivity the optical fiber density was measured in cryosections (12 μm) at different ages by ImageJ software. The quantification was performed in eight coronal serial sections per mouse on 8-bit images (grayscale). To measure the mean optical intensity the “Mean Gray Value” was determined. The respective immunoreactivity was measured by subtracting the mean gray value of the respective background from the mean gray value of dorsal striatum. The measurements were limited to the drawn region of interest and the same area was selected in all sections (0.05 mm^2^). The number of GFAP, pS6 and p62 and EM48 positive cells per microscopic field was counted in at least four independent paraffin sections per mouse in the dorsolateral striatal region. The EM48 signal area is calculated in μm^2^. We used the Quick LUT view to avoid the acquisition of images with under- and over-saturated pixels. The quantification and counting were performed blind to genotype and age.

### HPLC Analysis of Dopamine Content

The tissue levels of DA, 3,4-dihydroxyphenylacetic acid (DOPAC), homovanillic acid (HVA) and 3-methoxytyramine (3-MT), were measured using high-performance liquid chromatography with electrochemical detection (HPLC-EC). The concentrations of endogenous DA and its metabolites (DOPAC, HVA) were measured using HPLC-EC, according to a previously described method (Sikora et al., 2016). Briefly, the striatum was isolated by the adult mouse brain matrix (coronal slices, World Precision Instruments) in the 2 mm region comprised between Bregma +1.42 mm and −0.58 mm. After weighing the tissue samples were deep-frozen in dry ice and stored at −80°C until further use. Prior to analysis, the samples were homogenized in ice-cold 0.1 M HClO_4_ and were centrifuged at 10,000 *g* for 10 min at 4°C. The supernatant (5 μl) was injected into the HPLC system. The chromatography system consisted of an LC-4C amperometric detector with a cross-flow detector cell (BAS, IN, USA), an Ultimate 3000 pump (Thermoscientific, USA) and a Hypersil Gold analytical column (3 μm, 100 × 3 mm, Thermoscientific, USA). The mobile phase consisted of 0.1 M KH_2_PO_4_, 0.5 mM Na_2_EDTA, 80 mg/L sodium 1-octane sulfonate, and a 4% methanol, adjusted to pH 3.8 with an 85% H_3_PO_4_. The flow rate was 1 ml/min. The potential of a 3-mm glassy carbon electrode was set at 0.7 V with a sensitivity of 5 nA/V. The temperature of the column was maintained at 30°C. The Chromax 2007 program (Pol-Lab, Warszawa, Poland) was used for data collection and analysis. The external standard consisted of noradrenaline (NA), DA, 5-hydroxytryptamine (serotonin; 5-HT) and 5-hydroxy indole acetic acid (5-HIAA; SIGMA) at a concentration of 50 ng/ml.

### Statistical Analysis

All data are expressed as mean ± SEM. Two-tailed unpaired Student’s *t*-test was used for single comparisons. Multiple comparisons were performed either by one-way ANOVA or by two-way ANOVA with *post hoc* analyses as indicated in the figure legends (GraphPad Prism Software Inc.).

## Results

### Altered Primary Cilia Length in the Striatum but Not in Cortex of zQ175 Mice

**Figure 1:**
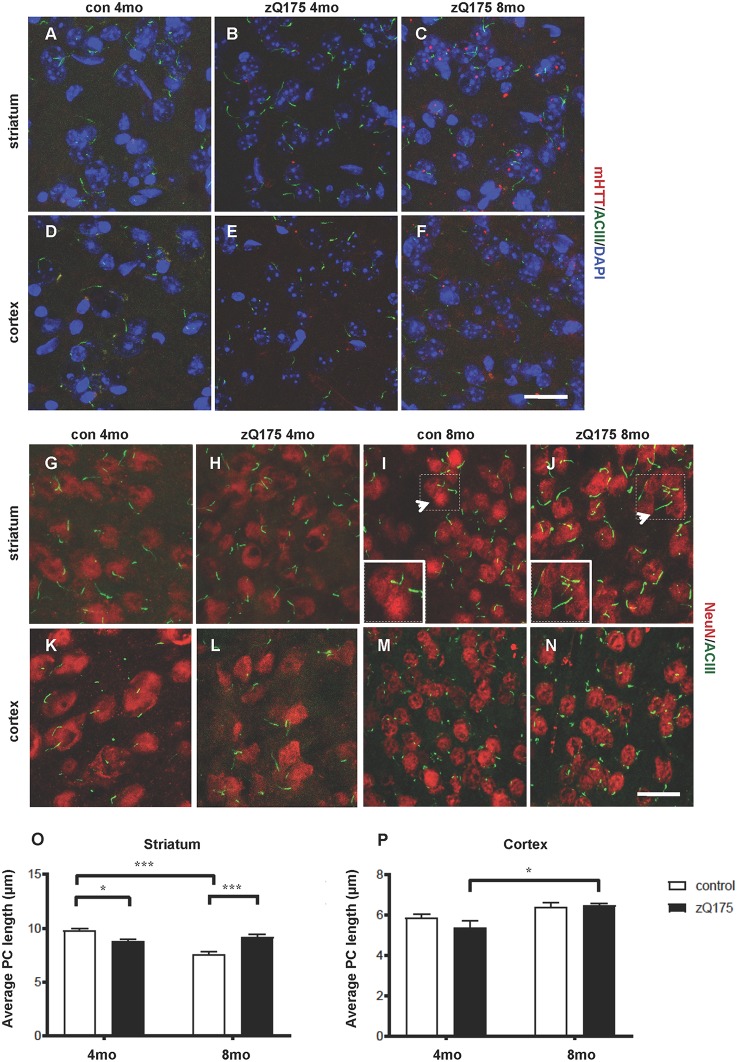
Context-specific changes in primary cilia (PC) length in the zQ175 Huntington’s disease (HD) mouse model are concomitant with mutant Huntingtin (mHTT) accumulation. **(A–F)** Representative confocal images of mHTT identified by the EM48 antibody (red) and of PC by adenylate cyclase III (ACIII), as a marker of PC (green) on cryosections from control and zQ175 mice in the dorsolateral striatum and cortex. Nuclei are visualized by 4′,6′-diamidino-2-pheylindol (DAPI) staining (blue). Scale bar: 25 μm. **(G–N)** Representative confocal images of immunofluorescent stainings on cryosections by NeuN, as a neuronal marker (red), ACIII (green), in the striatum **(G,J)** and cortex **(K,N)** to identify PC that protrudes from NeuN labeled neurons. Scale bars: 25 μm **(G–J, M,N)**, 12 μm **(K,L)**, 8 μm (insets, **I and J**). Arrows point to the area in the inset. **(O,P)** Diagrams showing the analysis of PC average length in striatal and cortical neurons at 4 and 8 months in control and zQ175 mice; *N* = 3, 5 control, and *N* = 4, 4 zQ175. Values represent means ± SEM. *p < 0.05, ***p < 0.0005, two-way ANOVA followed by Tukey’s *post hoc* test for multiple comparisons.

By IF and confocal analysis of brain sections, we observed the presence of sporadic mHTT inclusions at 4 months in the striatum. From 8 months mHTT accumulated mostly in the striatum but also in the cortex, in agreement with previous results (Carty et al., 2015; Figures 1A–F). To test the hypothesis that altered PC impacts HD neuropathology, we first investigated PC alterations in the heterozygous zQ175 knock-in model of HD. We measured the stage-specific alterations of PC length in both striatum and cortex at 4 and 8 months in control and zQ175 mice, focusing on cells labeled by the neuronal nuclei marker NeuN (Figures 1G–P). PC were identified by ACIII, a major PC marker in many regions of the adult mouse brain (Bishop et al., 2007). We found that PC in dorsolateral striatum of zQ175 mice were shorter than their littermate controls at 4 months, while they were longer than respective controls at 8 months (Figure 1O). Notably, PC length in the striatum of controls, decreased between 4 and 8 months, but this age-dependent decrease did not occur in the zQ175 mice (Figure 1O). Next, we analyzed the cell-specificity of this phenotype by measuring the average PC length in cortical cells (Figures 1K–N). Interestingly, cortical cells of zQ175 mice did not show changes in PC length at any of the considered stages (Figure 1P).

Hence, mHTT accumulation either directly or indirectly affects PC length in a stage- and region-specific fashion, suggesting that altered PC function might contribute to striatal vulnerability in HD.

### Conditional Ablation of the Ift88 Gene in Dopaminoceptive Neurons Leads to a Mild Increase of Dopamine Metabolite Levels in the Striatum

**Figure 2:**
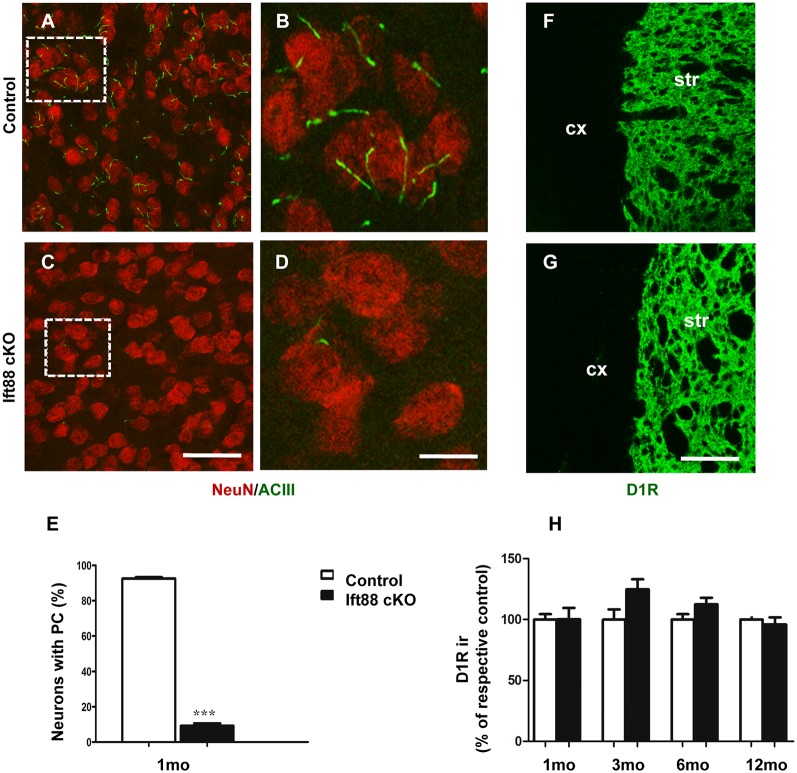
PC is dispensable for the maintenance of dopaminoceptive D1-receptor (D1R) expression in the striatum. **(A–D)** Representative images of immunofluorescent stainings showing PC stained with ACIII (green) and NeuN (red) in control and Ift88 cKO mice at 1 month. **(E)** Diagram shows the percentage of NeuN positive cells showing ACIII staining in control and Ift88 cKO at 1 month (*N* = 3). Error bars represent SEM, ***p < 0.001 based on two-tailed unpaired Student’s *t*-test. Scale bars represent 30 μm in **(A,C)** and 12 μm in **(B,D)**. **(F,G)** Examples of D1R immunostaining (green) on cryosections showing dorsolateral striatum in control and Ift88 cKO at 6 months; str, striatum; cx, cortex. **(H)** Diagram showing semi-quantitative analysis of D1R immunoreactivity (ir) in dorsolateral striatum at different ages; *N* = 3–5 controls, *N* = 3–5 Ift88 cKO. Values represent means ± SEM. Scale bar in **(F,G)**: 100 μm. No significant differences with respective controls by unpaired Student’s *t*-test.

To investigate the effects of PC loss on HD neuropathology, we first generated inducible mutant mice conditionally lacking *Ift88*, a gene encoding a microtubular component essential for the formation of the PC (Haycraft et al., 2007). These mice are characterized by the expression of Cre recombinase under the control of dopaminoceptive D1-receptor (D1R; Lemberger et al., 2007; Figures 2A–D). The conditional ablation of the *Ift88* gene resulted in D1R:Cre;*Ift88*^flox/flox^ (abbreviated as Ift88 cKO) mice lacking PC in most of the striatal neurons stained for neuronal nuclei (NeuN; Figure 2E). The *Ift88* cKO mice did not show any gross abnormalities. We monitored changes in body weight (g) over time and we found evidence of increased weight in the Ift88 cKO at 1 year (eight male control, 34.4 ± 6.1 vs. six male mutants, 44.9 ± 4.9; *p* = 0.004 two-tailed Student’s *t-test*). This conditional model enabled us to mimic a condition of loss of PC in dopaminoceptive neurons to identify their context-specific function. Next, we analyzed D1R expression as a read-out of the survival of positive neurons in control and Ift88 cKO at different ages (1, 3, 6, and 12 months; Figures 2F–H). To investigate the impact of PC loss on neuronal survival, we analyzed the immunoreactivity of D1R in dorsolateral striatum at different ages in control and *Ift88* cKO mice by IF and semi-quantitative analysis of D1R mean signal intensity upon confocal imaging (Figure 2H). This analysis showed no significant differences in D1R immunoreactivity at any of the considered ages in control and *Ift88* cKO mutant mice, if at all a tendency to higher D1R immunoreactivity, suggesting that PC is not required for survival of D1R MSNs under basal conditions.

**Figure 3:**
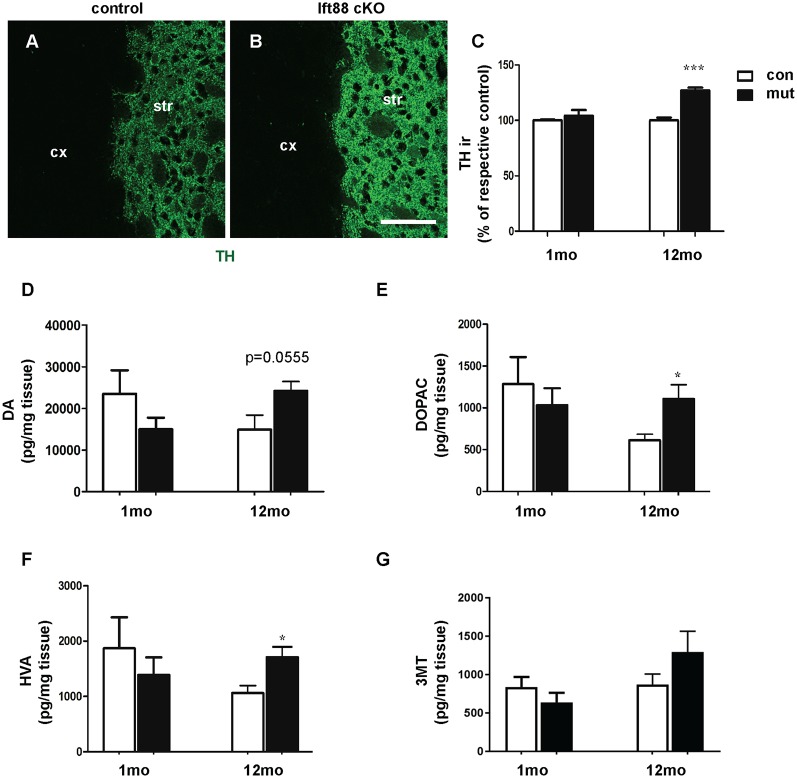
Loss of striatal PC results in a mild increase of dopamine (DA) metabolites in the striatum. **(A,B)** Examples of tyrosine-hydroxylase (TH) immunofluorescence (IF), as a marker for dopaminergic projections, in cryosections from the dorsolateral striatum of control and Ift88 cKO mice (1-year-old). Scale bar: 100 μm. **(C)** Semi-quantitative analysis of TH immunoreactivity (ir) at 1 and 12 months; *N* = 5 per group. **(D–G)** Levels of DA and its major metabolites [3,4-dihydroxyphenylacetic acid (DOPAC), homovanillic acid (HVA), 3-methoxytyramine (3-MT)] in the striatum by HPLC-EC; *N* = 5 per group. Values represent means ± SEM. *p < 0.05, ***p < 0.001 by unpaired Student’s *t*-test with respective controls.

To further characterize the phenotype of the *Ift88* cKO, we investigated dopaminergic input to dopaminoceptive striatal neurons (Figure 3). To this end, we focused on young (1 month) and older (1 year) mice. We measured tyrosine-hydroxylase (TH) immunoreactivity by IF (Figures 3A,B). Interestingly, TH immunoreactivity increased in the mutant at 1 year (Figure 3C). Next, we asked whether the loss of PC in D1R neurons affects DA content in the striatum (Figures 3D–G). High performance liquid chromatography followed by electrochemical detection (HPLC-EC) showed that 1-year-old *Ift88* cKO mutant mice are characterized both by a tendency to increased levels of total DA content in the striatum as well as by a mild but significant increase of the two most important DA metabolites DOPAC, and HVA (Figures 3D–F).

These observations indicate subtle PC-dependent crosstalk between PC-depleted dopaminoceptive neurons and dopaminergic neurons to cope with a potentially altered dopaminergic neurotransmission.

### Loss of PC in the zQ175 HD Mice Results in mTOR Activation and in Larger mHTT Nuclear Inclusions

**Figure 4:**
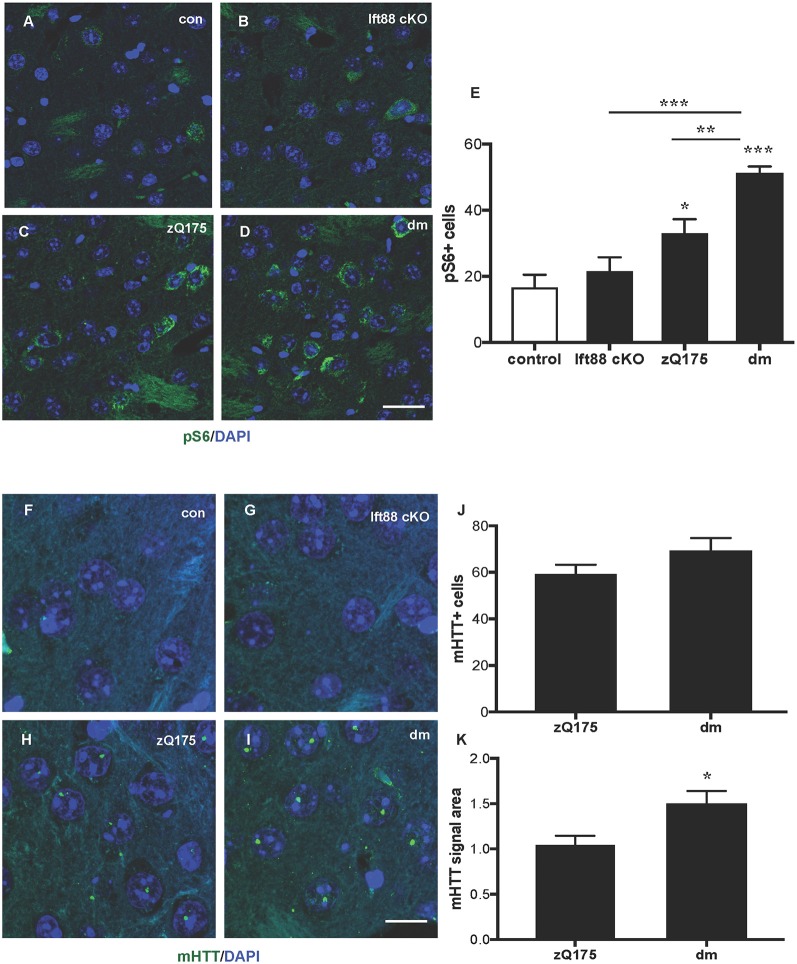
Impact of PC loss in medium spiny neurons (MSNs) of zQ175 HD mice on the mechanistic target of rapamycin (mTOR) pathway and mHTT nuclear inclusions. **(A–D)** Representative confocal images of phopsho-S6 (pS6) IF (green) and DAPI staining (blue) on paraffin sections showing dorsolateral striatum in control, Ift88 cKO, zQ175, and dm at 8 months. Scale bar: 25 μm. **(E)** Quantification of the pS6 positive cells expressed as mean values of the number of counted cells per microscopic field. Control vs. zQ175 *p < 0.05; control vs. dm ***p < 0.0001 Ift88 cKO vs. dm ***p < 0.001, zQ175 vs. dm **p < 0.01 by one-way ANOVA followed by Dunnett’s *post hoc* test for multiple comparisons. Values represent means ± SEM. pS6: control (*N* = 5), Ift88 cKO (*N* = 5), zQ175 (*N* = 6) and dm (*N* = 5) mice. **(F–I)** Representative confocal images of mHTT immunostaining (green) by EM48 antibody and DAPI (blue) on paraffin sections from dorsolateral striatum in control, Ift88 cKO, zQ175, and dm at 8 months. Scale bar: 10 μm. **(J,K)** Quantification of the EM48 positive cells expressed as a percentage of DAPI positive cells and of the mean EM48 signal area (in μm^2^) per microscopic field. Values represent means ± SEM. *p < 0.05 (*p* = 0.019) based on two-tailed unpaired Student’s *t*-test; zQ175 (*N* = 8) and dm (*N* = 6) mice.

To study the impact of PC loss on HD neuropathology, we generated zQ175 heterozygous mice lacking PC in striatal but not in cortical neurons in an *Ift88* cKO background (double mutants, dm; Supplementary Figure S1). To establish whether PC loss is toxic in combination with the zQ175 mutation and whether the combination of these mutations results in a more severe HD phenotype, we analyzed control, *Ift88* cKO, zQ175, and dm at 8 months (Supplementary Figure S2 and Figure 4). We found no significant difference in the number of GFAP positive cells, as a marker of astrogliosis, associated with neurodegeneration, suggesting that there is no induction of massive degeneration at least until this stage (Supplementary Figures S2A–D,I). To monitor the clearance of protein aggregates, we quantified changes in the number of sequestosome 1 (SQSTM1/p62) protein immunopositive cells in the dorsolateral striatum by IF in the four experimental groups above (Supplementary Figures S2E–H,J). p62 is a cargo-binding protein associated with proteotoxic stress (Johansen and Lamark, 2011). As shown in Supplementary Figures S2G,H, the localization of p62 in the zQ175 and in the dm mice was intra-nuclear rather than cytoplasmic, as in controls and *Ift88* cKO, however, the number of p62 positive cells was comparable between all groups. Next, we investigated mTOR kinase activity by IF based on the levels of one of its targets, phosphorylated ribosomal protein S6 (phospho-S6, pS6; Figures 4A–E). Interestingly, zQ175 and dm mice showed a higher number of pS6 positive cells in comparison to controls, and this was significantly higher in the dm in comparison to the zQ175 (Figure 4E), suggesting that PC loss in the zQ175 mice promotes mTOR activation. Moreover, we analyzed mHTT nuclear inclusions that are absent in controls and Ift88 cKO, and visible in the zQ175 and dm (Figures 4F–I). At the age examined (8 months), the number of mHTT positive cells was similar in the zQ175 and dm mice (Figure 4J). The area of the mHTT signal in the dm is ca. Thirty percent larger in comparison to the zQ175 mice, indicating that PC limit mHTT accumulation in this model (Figure 4K).

## Discussion

In the present study, we addressed the following three questions: (1) whether PC is specifically altered in different neuronal types and HD stages; (2) what are the consequences of PC disruption in striatal neurons for their survival; and (3) to what extent these contribute to HD neuropathology in a neuronal population vulnerable in HD such as the striatum. We showed that PC length is affected in the striatum in association with mHTT accumulation. PC disruption in MSN does not affect neuronal survival. Subtle compensatory mechanisms might be activated by dopaminergic neurons in response to PC loss on dopaminoceptive neurons; however, a detailed characterization awaits future experiments. PC disruption in striatal neurons of an HD mouse model results in increased mTOR activation and larger mHTT nuclear inclusions, suggesting that PC is required in a pathological context.

Changes in PC length have been reported in previous animal and cellular models expressing mHTT; however, this is the first study focusing on PC pathology in the striatal neurons that are pathologically affected in HD and addressing the impact of loss of PC in a progressive mouse model of HD. Here, we identified changes in PC length that are concomitant with mHTT accumulation: PC length is altered in striatal but not in cortical neurons in the zQ175 mice. Previous work has indicated that HTT is important for the transport of proteins required for ciliogenesis (Keryer et al., 2011). Ciliogenesis is a tightly regulated process, and it is crucial for its proper function that PC is produced in the right size and time (Avasthi and Marshall, 2012). Given the time- and cell-specific impact of mHTT on PC, it will be important to better understand how differences in mHTT state differentially modulate PC maintenance.

Interestingly, various HD models show altered levels of DA and its metabolites (Smith et al., 2014; Koch and Raymond, 2019), and lack of dopaminergic inputs results in longer PC in the striatum (Miyoshi et al., 2014). Hence changes of DA input in the zQ175 mice might alter PC length. Because DA receptors are located in the PC membrane (Marley and von Zastrow, 2010; Domire et al., 2011; Leaf and Von Zastrow, 2015), it will be important to investigate striatal synaptic transmission, and motor and psychiatric phenotypes in the HD mice lacking PC. Future studies should address the behavioral implications of the mild increase in DA metabolite levels and increased TH expression in the striatum. These studies might help to understand the impact of current DA-based symptomatic HD treatments on PC physiology and homeostasis.

The generation of tools enabling targeting of PC function independently of changes in their structural integrity might be necessary to define subtle regulatory functions. The *Ift88* cKO approach, in which both PC function and structure are affected, does not allow us to dissect out the specific role of PC signaling from PC structure, however, it does allow us to identify cell-autonomous effects as well as non-cell autonomous responses to ciliary impairment. In aged homozygous zQ175 mice mTOR activity increases (Abd-Elrahman and Ferguson, 2019). We found in the zQ175 and in the dm mice increased phospho-S6 positive cells. Hence, another possible mechanism to explain changes in PC length might be linked to a block of autophagy by mTOR activation (Pampliega et al., 2013). Together, it will be important to analyze older zQ175 and dm mice. Conditional ablation of Ift88 in neural progenitors by the Nestin-Cre transgenic line revealed a significant increase in mTOR pathway activity and phospho-S6 ribosomal protein levels, resulting in hydrocephaly at late embryonic stages (Foerster et al., 2017). In the D1R-Cre driven conditional Ift88 knock-out, phospho-S6 does not increase in comparison to controls. These results indicate the cell-type, developmental stage and age-specific function of PC alterations, and support the need to further address this question in a mature and aging brain.

Diseases primarily caused by ciliary dysfunctions are commonly referred to as ciliopathies. As of yet, we cannot ascribe HD among the primary ciliopathies, nevertheless, PC structure and function are altered in association with a more severe phenotype. Notably, the role of PC in neuronal homeostasis, and in the control of the cellular stress response signaling cascades is emerging in other neurodegenerative disorders. PC pathology has been described for example in Alzheimer’s disease (AD), in Parkinson’s disease (PD) and in spinocerebellar ataxias, another polyglutamine disease, although distinct mechanisms are probably involved (Steger et al., 2017; Bowie et al., 2018; Dhekne et al., 2018; Vorobyeva and Saunders, 2018). Interestingly, PC were elongated in the hippocampus of the APP/PS1 mouse models of AD compared with wild-type mice, and serotonin 5-HT6 receptors playing a critical role in AD development regulate the morphology and function of neuronal PC (Hu et al., 2017). Moreover, in mouse models of PD expressing mutant LRRK2 R1441C, PC was affected in cholinergic neurons while the overall ciliation of neurons in the striatum was not significantly different from wild type (Steger et al., 2017; Dhekne et al., 2018). Nevertheless, defective ciliogenesis in striatal cholinergic neurons might impair a protective mechanism involving non-cell autonomous Sonic hedgehog between cholinergic and dopaminergic neurons (Gonzalez-Reyes et al., 2012). The Spinocerebellar ataxia type 11-associated mutation of the serine/threonine kinase Tau tubulin kinase 2 dominantly interferes with ciliogenesis and cilium stability (Bowie et al., 2018). Shortened primary cilium length and dysregulated Sonic hedgehog signaling were also reported in Niemann-Pick type C1 (NPC1) disease, a neurodegenerative lysosomal storage disorder caused by mutations in the NPC1 gene (Canterini et al., 2017).

In summary, although we obtained similar mean PC length as previously reported for striatum and cortex in mice (Sipos et al., 2018), a systematic quantitative comparison of PC marker expression and length in various neurodegenerative disease models and in human tissues will benefit from the use of automatized segmentation approaches (Vorobyeva and Saunders, 2018), and high content automated image acquisition. These future studies will be important to determine in other disease models which cells and tissues display the cilia defects and at what stages, and try to understand when, whether and how those changes lead to specific neuronal loss in the brain.

## Data Availability Statement

All datasets generated for this study are included in the article/Supplementary Material.

## Ethics Statement

Procedures involving animal care were approved by the Committee on Animal Care and Use (Regierungspräsidium Karlsruhe, Germany) in accordance with the local Animal Welfare Act and the European Communities Council Directives (2010/63/EU and 2012/707/EU; Ref. number: G-252/17).

## Author Contributions

RM, GK, KK, and RP: data acquisition. RM, GK, JK, A-EW, KT, and RP: data analysis and interpretation. RP: study concept and design, drafting of the manuscript. All authors revised the submitted manuscript.

## Conflict of Interest

The authors declare that the research was conducted in the absence of any commercial or financial relationships that could be construed as a potential conflict of interest.

## Abbreviations

ACIII: adenylate cyclase III
AD: Alzheimer’s disease
DA: dopamine
DAPI: 4′,6′-diamidino-2-pheylindol
DOPAC: 3,4-dihydroxyphenylacetic acid
D1R: dopaminoceptive D1-receptor
GFAP: glial fibrillary acidic protein
HD: Huntington’s disease
HPLC-EC: High-performance liquid chromatography-electrochemical detection
5-HT: 5-hydroxytryptamine
HVA: homovanillic acid
IF: immunofluorescence
IFT-B: intraflagellar transport B
IHC: immunohistochemistry
mHTT: mutant Huntingtin
MSNs: medium spiny neurons
mTOR: mechanistic target of rapamycin
NeuN: neuronal nuclei
NPC1: Niemann-Pick type C1
PC: primary cilia
PD: Parkinson’s disease
PFA: paraformaldehyde
phospho-S6: phosphorylated ribosomal protein S6
p62/SQSTM1: p62/sequestosome 1
TH: tyrosine-hydroxylase.
