# Supplementary material for: Targeted Depletion of Primary Cilia in Dopaminoceptive Neurons in a Preclinical Mouse Model of Huntington’s Disease
Source: Front Cell Neurosci. 2019 Dec 20;13:565. doi: 10.3389/fncel.2019.00565 (PMC6936315; doi:10.3389/fncel.2019.00565)
Supplement: Supplementary file 1 [file Table_1.docx]

**Brief Research Article**

**Title:**

**Targeted depletion of primary cilia in dopaminoceptive neurons in a preclinical mouse model of Huntington’s disease**

Rasem Mustafa, Grzegorz Kreiner, Katarzyna Kamińska, Amelia-Elise J. Wood, Joachim Kirsch, Kerry L. Tucker, Rosanna Parlato

**Suppl. Figure Legends**

**Suppl. Figure 1: Targeted disruption of PC in striatal but not in cortical neurons of Ift88 cKO and dm mice.** (A-F) Representative confocal images of immunofluorescent stainings on cryosections by NeuN (red) and ACIII (green) in striatum and cortex to identify PC that protrude from NeuN labeled neurons. Scale bar: 25µm. (G) Diagrams comparing the PC number in striatum and cortex of control (N=5), Ift88 cKO (N=4) and dm (N=4) mice at 8 months. Values represent means ± SEM. ***, p < 0.001, one-way ANOVA followed by Dunnett’s post-hoc test for multiple comparisons.

**Suppl. Figure 2: PC loss in the zQ175 HD mice does not increase astrogliosis, and p62 positive cells.** (A-D) Representative confocal images of GFAP immunofluorescence (green) and DAPI staining (blue) on paraffin sections showing dorsolateral striatum in control, Ift88 cKO, zQ175, and dm at 8 months. Scale bar: 100µm. (E-H) Representative confocal images of p62 immunostaining (green) and DAPI (blue) on paraffin sections from dorsolateral striatum in control, Ift88 cKO, zQ175, and dm at 8 months. Scale bar: 25µm. (I, J) Quantification of the GFAP and p62 positive cells expressed as mean values of the number of counted cells and as percentage of DAPI positive cells per microscopic field. Values represent means ± SEM. No significant differences by one-way ANOVA; GFAP: control (N=5), Ift88 cKO (N=5), zQ175 (N=6) and dm (N=5) mice and p62: control (N=6), Ift88 cKO (N=5), zQ175 (N=8) and dm (N=6) mice.
