# Supplementary figures and images for: Targeted Depletion of Primary Cilia in Dopaminoceptive Neurons in a Preclinical Mouse Model of Huntington’s Disease
Source: Front Cell Neurosci. 2019 Dec 20;13:565. doi: 10.3389/fncel.2019.00565 (PMC6936315; doi:10.3389/fncel.2019.00565)

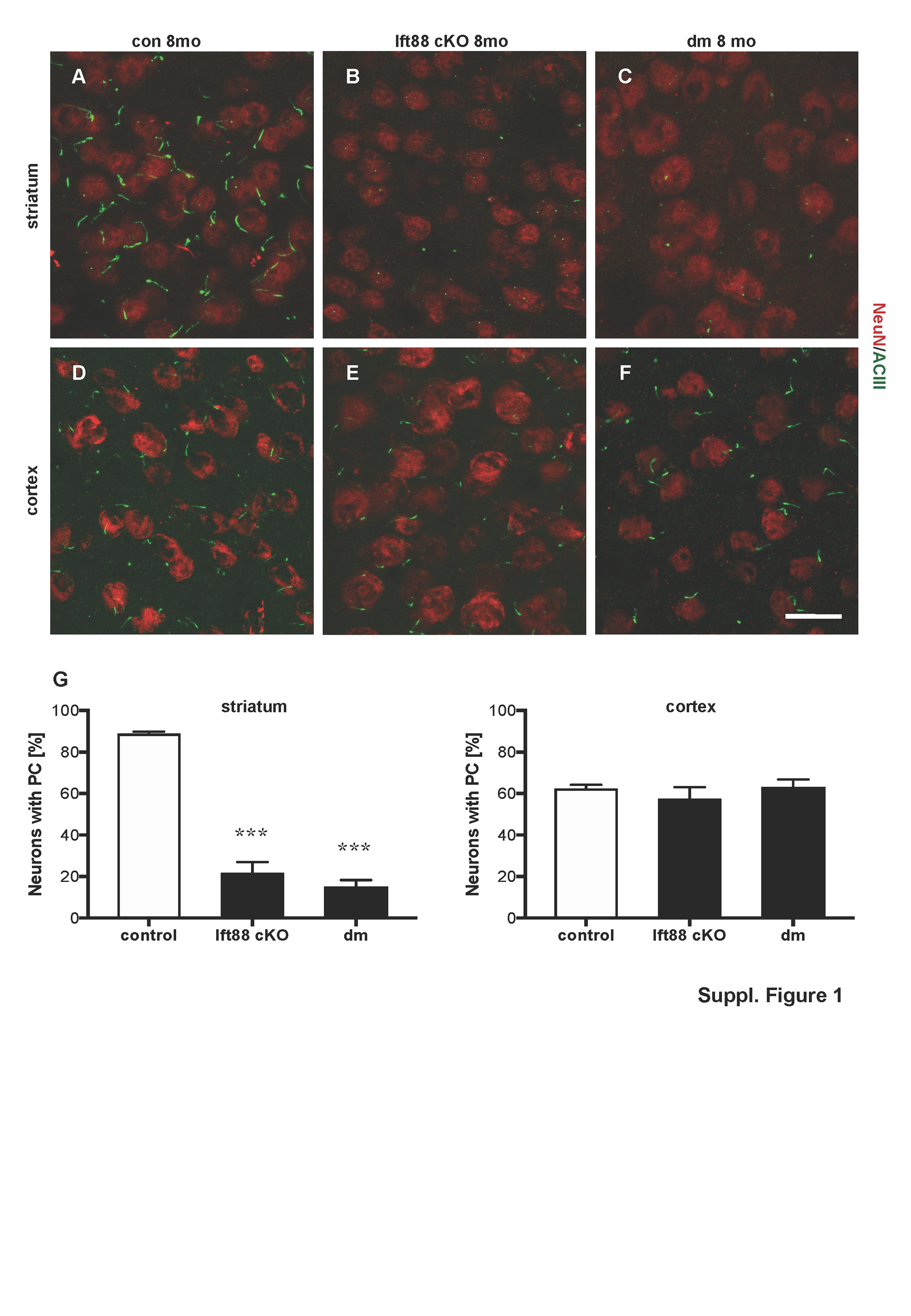

Supplement: Supplementary file 2 [file Image_1.TIFF]

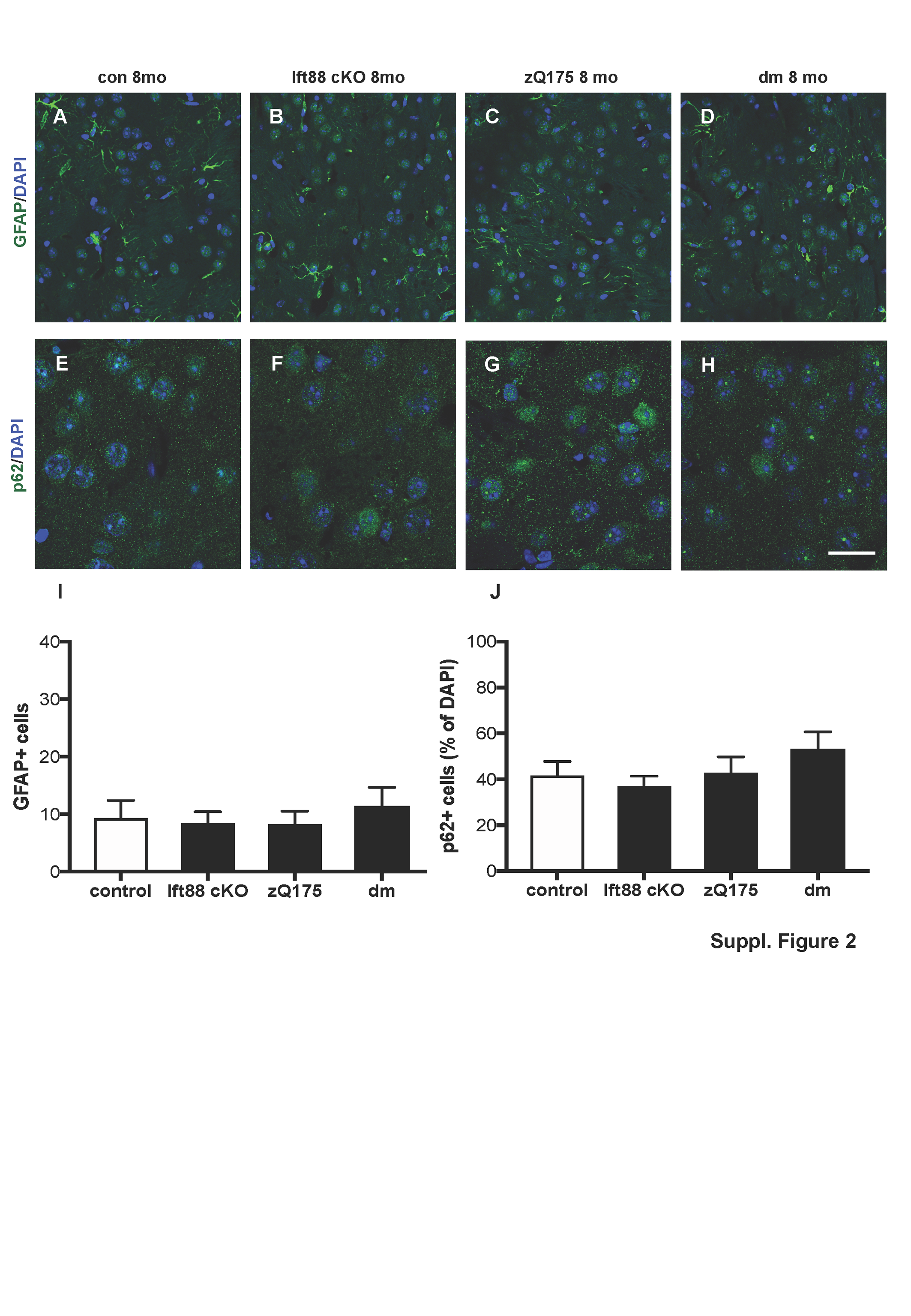

Supplement: Supplementary file 3 [file Image_2.TIFF]
